# Conservation of the role of *INNER NO OUTER* in development of unitegmic ovules of the Solanaceae despite a divergence in protein function

**DOI:** 10.1186/s12870-016-0835-z

**Published:** 2016-06-27

**Authors:** Debra J. Skinner, Ryan H. Brown, Robert K. Kuzoff, Charles S. Gasser

**Affiliations:** Department of Molecular and Cellular Biology, University of California, Davis, Davis, CA 95616 USA; Present address: US Patent and Trademark Office, 400 Dulany St, Alexandria, VA 22314 USA; Present address: Department of Biological Sciences, University of Wisconsin-Whitewater, Whitewater, WI 53190 USA

## Abstract

**Background:**

The *INNER NO OUTER* (*INO*) gene is expressed in the outermost cell layer of the outer integument of bitegmic ovules and is essential for this organ’s growth. The role and cross-species functional conservation of *INO* orthologs were examined in members of the Solanaceae, which have unitegmic ovules. Unitegmy has evolved several times in disparate angiosperm lineages. *INO* expression has been observed in the outermost cell layers of all examined unitegmic ovules, but the functional role of *INO* in unitegmic ovules has not previously been evaluated.

**Results:**

*INO* orthologs were unambiguously identified in tobacco and tomato by sequence homology. Expression of the tomato *INO* gene was limited to the outer cell layer of the single integument indicating that this single integument has properties of the outer integument. Expression occurred only after integument initiation, later than observed in ovules of other examined angiosperms. Virus-induced knock-down of expression of the *INO* ortholog in tobacco inhibited growth of the outer cell layer of the integument leading to a decrease in both integument extension and curvature of the ovule. The altered ovules closely resemble those of the *aberrant testa shape* (*ats*) *ino* mutant combination in Arabidopsis where we see the effect of the *ino* mutation on a single fused integument produced by the *ats* mutation. Despite significant sequence identity and similar expression patterns, the tomato *INO* coding region was not able to complement the Arabidopsis *ino* mutant.

**Conclusions:**

The similarity of effects of *ino* mutations on the unitegmic ovules of tobacco and the fused integuments of the Arabidopsis *ats* mutant show that: 1) *INO* orthologs play the same role in promoting integument growth in ovules of tobacco and Arabidopsis; and 2) the unitegmic ovules of tobacco (and hence other solanaceous species) are most likely the result of a congenital fusion of two ancestral integuments. Our results further indicate that INO has a conserved role in growth of the outermost cell layer of integuments. The curvature of solanaceous ovules is driven by unequal growth of the outer layers of the single integument that likely correspond to an ancestral outer integument.

**Electronic supplementary material:**

The online version of this article (doi:10.1186/s12870-016-0835-z) contains supplementary material, which is available to authorized users.

## Background

Among seed plants, ovule structure is largely conserved in comprising three main structures: the nucellus, site of megagametophyte development; the integument(s), sheath(s) of cells that surround the nucellus; and the funiculus, a stalk providing a physical and vascular connection to the placenta. In the angiosperms, significant variation in ovule form occurs in the number and thickness of integuments and curvature of the ovule [1]. The earliest diverging angiosperm lineages are largely bitegmic [2, 3] and anatropous (curved toward the placenta), two characters that are likely to be intimately connected [4]. Ovule curvature is often caused by asymmetric integument growth, usually of the outer integument as seen, for example, in bitegmic ovules of Arabidopsis. The outer integument initiates in a semi-annular pattern around the chalaza and continues to grow only on the gynobasal (toward the base of the gynoecium) side of the ovule. In Arabidopsis outer integument initiation and growth require the activity of the *INNER NO OUTER (INO)* gene, expressed at the site of integument initiation and persisting in the outer layer of the growing outer integument [5, 6]. Arabidopsis mutants lacking a functional copy of *INO* fail to produce an outer integument. Additionally, in the *superman* mutant symmetric expression of *AtINO* around the chalaza leads to development of symmetric outer integument, and symmetric largely orthotropous ovules.

*INO* expression has been studied in a range of taxa, including early diverging bitegmic angiosperms such as *Cabomba caroliniana* (*CcINO*) (Nymphaeales) and *Annona squamosa* (Magnoliales) and in these early lineages, the expression of *INO* in the outermost cell layer of the outer integument is conserved [7, 8]. Moreover, a unitegmic *A. squamosa* mutant lacks the *INO* gene and the outer integument is absent, suggesting that the role of *INO* in outer integument growth was established early in the angiosperm lineage [7]. Thus, *INO* expression can be used as a practical marker for the outer integument that is useful in understanding the nature and evolution of integuments in angiosperms.

Where such genes show conservation of expression and function across diverse taxa, it is informative to ask how that function has been maintained at the level of regulation of expression and the activity of the protein. Cross species complementation can indicate whether proteins have diverged in their targets and activity. Similar experiments and examination of alignments between promoters can indicate regions that retain a shared regulatory function.

Unitegmy has arisen from the ancestral bitegmic state in several angiosperm lineages through different proposed mechanisms such as fusion of the two integuments, loss of an integument, or integumentary shifting [9]. Unitegmy is a shared character of the euasterids, indicating a single origin of this character for this clade [10], but the derivation of this morphology in euasterids has not yet been elucidated. The closest relatives of the euasterids that retain bitegmic ovules are in the Ericales grade, the basal lineages in the broader asterid clade, where several genera have bitegmic ovules [10]. We previously examined species with unitegmic, bitegmic and intermediate ovules in *Impatiens*, a genus within the Ericales [11]. We found that integument fusion was the cause of derived unitegmy in this genus, and that anatropous curvature of all of these ovules derived primarily from curvature of the present, or ancestral outer integument [11].

Ovule development is nearly invariant in the euasterids, even in members of the most distantly related clades [10, 12], such as *Aster* and *Solidago* in the Asterales [13], Antirrhinum [14] in the Lamiales, and *Solanum lycopersicum* (tomato) [15, 16] in the Solanales. Ovule development among members of the Solanales such as as tomato [15, 16], tobacco [17] and petunia [18, 19], is sufficiently invariant that a description of one species can suffice for all three. In tomato, rows of ovule primordia arise on the central placenta during floral stage 8, and the megaspore mother cell differentiates, with one layer of nucellar cells covering the megaspore [15, 16]. As the megaspore becomes visible, the ovule primordium grows asymmetrically, leading to a slightly curved primordium. The integument primordium arises just below the level of the megaspore during stage 11 on the thickened side of the ovule. The integument grows asymmetrically to cover the nucellus and at maturity the ovule is fully curved with the micropyle adjacent to the placenta. The funiculus remains short throughout ovule development. Tomato is a good research model for euasterids due to the recent sequencing of the tomato genome [20] and the existence of many phenotypic variants (TGRC.ucdavis.edu), while tobacco has established methods for gene function evaluation through Virus Induced Gene Silencing (VIGS, [21]). In this study we examined the role of the *INO* gene in the unitegmic solanaceous species tomato and tobacco during integument development by assessing gene expression, function and sequence conservation.

## Results

### Isolation of solanaceous *INO* sequences

The tomato (*Solanum lycopersicum*) genome includes nine YABBY genes [20]. In a phylogenetic analysis of these genes, together with representative genes from other angiosperms, five clades were identified [22, 23], (Additional file 1: Figure S1), consistent with prior analyses of the gene family in other angiosperms [8, 24]. While there was only a single tomato gene in the *INO* clade (*SlINO*), the other sub-families of YABBY genes: *CRC*, *YAB2*, *YAB3/FIL*, and *YAB5* were each represented by two genes in tomato (Additional file 2: Table S1). To facilitate characterization, *SlINO* cDNA was isolated from a tomato pistil cDNA library [25] by hybridization with the Arabidopsis *INO* cDNA. This coding sequence confirmed the sequence of the predicted protein identified as SlINO (Solyc05g005240) in the tomato genome (Solgenomics.net, [22]). Using *SlINO* cDNA as a probe, a genomic clone of SlINO that included the first four exons and the 5’flanking region up to the adjacent predicted gene was isolated from a genomic library [26].

*Nicotiana benthamiana* is a recent allotetrapoid and includes two paralogous copies of each of the YABBY genes (Solgenomics.net, [27]). Consistent with this, two *INO* homologs were identified by homology searches of the draft sequence and predicted genes at solgenomics.net: Niben101Scf09599g00012.1 (*NbINO1*) and Niben101Scf04287g04009.1 (*NbINO2*). *NbINO1* appears to be incorrectly annotated, based on alignment with *SlINO* and *AtINO*, and is more likely to be as shown in Additional file 3: Figure S2. The two encoded INO proteins are 95 % identical to each other and are 76 % identical and 84 % similar to SlINO along their aligned length, and clearly group with other INO proteins in phylogenetic analysis (Additional file 1: Figure S1). Polymerase chain reaction was used to isolate a partial genomic clone of *NbINO1* for use in VIGS.

### Conservation of *INO* expression in tomato

**Fig. 1:**
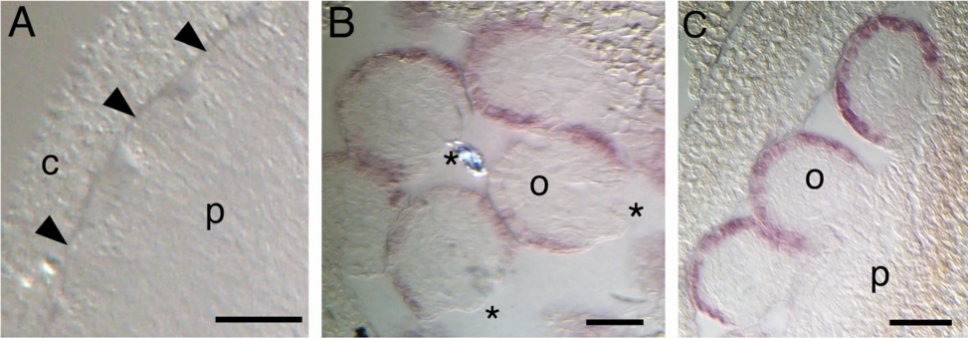
Integument specific expression of *SlINO.* **a**-**c** *In-situ* hybridization with a labeled *SlINO* antisense RNA probe in tomato ovules. Purple stain indicates hybridization to *SlINO* mRNA. **a** Longitudinal section of stage 8 carpel with ovule primordia (arrowheads) prior to integument initiation showing no hybridization signal. **b** Cross-section of stage 14 ovules with exposed nucelli (*) showing hybridization signal in outermost cell layer of the integument. **c** Stage 16 ovules show the specificity of the *SlINO* expression pattern, with no hybridization in placenta, funiculus or inner cell layers of the ovule. arrowhead, ovule primordium; *, nucellus; o, ovule; p, placenta. Scale bars are 40 μm

A feature of characterized *INO* genes is specific expression in the outer layer of the outer integument or single integument of ovules, with this pattern being conserved among eudicots like Arabidopsis [6], *Impatiens* [11] and *Prunus* [28] and members of early branching groups such as the Magnoliales (*Annona* [7]) and Nymphaeales (*Cabomba* [8]). Expression profiling showed expression of the *S. pimpinellifolium* (wild tomato LA1589) *INO* ortholog to be limited to flowers during the period of ovule development and two days post anthesis, with the peak of expression at anthesis [22, 29]. *In situ* hybridizations using the *SlINO* cDNA were performed on tomato carpels (Fig. 1a-c). *SlINO* transcripts were only detected in ovules, where they were confined to the outermost layer of the single integument, and were not observed in early ovule primordia. These results suggest that in the unitegmic tomato, the integument has characteristics of the outer integument, similar to results seen in unitegmic *Impatiens* and *Prunus* species [11, 28].

**Fig. 2:**
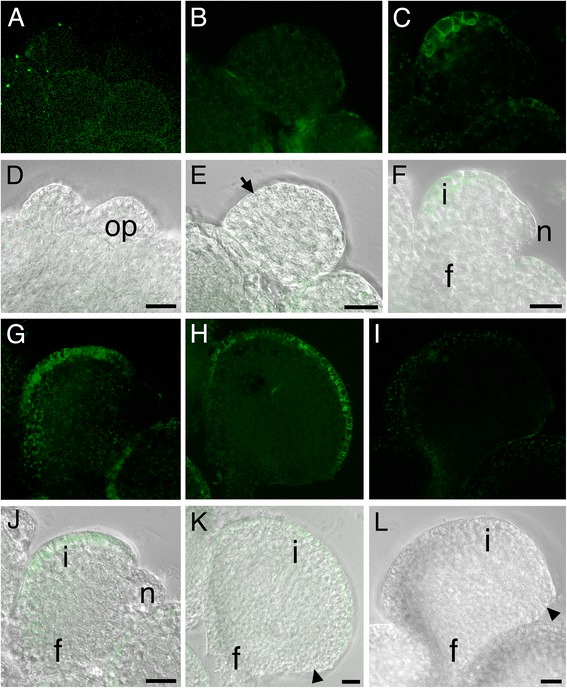
Timing and specificity of a putative *SlINO* promoter directing expression of an *SlINO-GFP* transgene**.** Confocal images (**a**-**c**, **g**-**i**) overlaid on corresponding DIC images (**d**-**f**, **k**-**l**) of tomato ovules. Control ovule with no transgene is shown in **i**, **l**. In this ovule the visible fluorescent dots are likely to be auto-fluorescence from plastids. All other images are from plants containing a P-*SlINO::SlINO-GFP* transgene. No GFP signal is present in stage 8 ovule primordia prior to integument initiation (**a**, **d**) or at stage 10, when asymmetric thickening of the primordium (arrow) signals emergence of the integument (**b**, **e**). GFP localized in the outermost cell layer is visible in the newly emerged integument primordium in stage 11 (**c**, **f**). This pattern continues as the integument grows asymmetrically to cover the nucellus in stage 13 (**g**, **j**) and form the micropyle in the mature ovule at stage 18 (**h**, **k**). Expression is low or absent in the cells at the distal tip of the integument (**c**, **g**, **h**).op, ovule primordium; f, funiculus; i, integument; n, nucellus; o, ovule; p, placenta; arrowhead, micropyle. Scale bars are 20 μm

For further expression analysis and for heterologous expression, a putative promoter region including 2257 bp of 5’ flanking and untranslated sequence (P-*SlINO*) extending to the next upstream gene was isolated from the genomic clone. This region was used to drive transcription of green fluorescent protein (GFP) fusion genes in tomato (Fig. 2). P-*SlINO*::*SlINO*:*GFP* gave results similar to the *in situ* hybridization. Expression was detected only in the outer cell layer of the integument and was first visible after the emergence of the integument (Fig. 2b, c). Expression was low or absent from the few cells at the distal tip of the integument (Fig. 2 all stages), and also absent from integument cells on the side of the ovule opposite from the side on which most growth occurs (Fig. 2j, k). Expression in the outer layer of the integument remained high in mature ovules at anthesis (Fig. 2k) and continued to be visible through the onset of fruit development (Additional file 4: Figure S3). These results showed that the 5’-flanking and 5’-untranslated regions of *SlINO* contained sufficient information to duplicate the *in situ* results. The expression pattern is similar to that seen in Arabidopsis [6], *Impatiens* [11], *Prunus* [28], *Annona* [7] and *Cabomba* [8], differing from Arabidopsis in that *SlINO* expression was not detected prior to integument initiation, was maintained for longer during development, and the signal was spread over nearly the entire surface of the integument, rather than being concentrated toward the base of the integument.

### Conservation of role of INO in ovule development

**Fig. 3:**
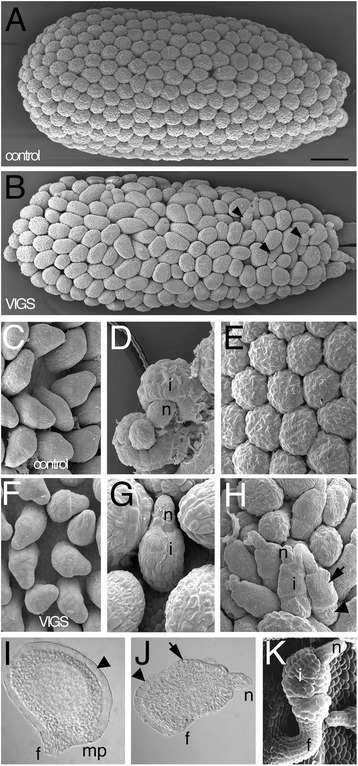
VIGS knock-down of *NbINO* activity in *N. benthamiana* ovules. **a** Ovary of control carpel shows round and symmetrical ovules packed tightly on the placenta. **b** In a representative knock-down carpel the ovules are variable in shape and some are incompletely formed (examples indicated with arrowheads). Ovules shown in **c**-**e**, **i** are from control plants and **f**-**h**, **j** are knock-down ovules at similar stages. In **c** and **f** the integument appears as a thickening of the chalazal area and causes bending of the ovule primordium. The integument grows asymmetrically (**d**) while in knock-down ovules (**g**) growth of the integument is reduced. At maturity (**e**) the nucellus is no longer visible and ovules appear isodiametric. Knock-down ovules (**h**) are variable in shape and some are aberrant, with the nucellus protruding from a variable integument. The large cells of the outer cell layer of the integument (arrowhead) visible in **i** are only covering the basal part of the integument in **j**. Smaller dense cells form the outermost layer of the distal part of the integument (arrow). **k** Arabidopsis *ats ino* double mutant ovule resembles the VIGS knock-down tobacco ovules. i: integument; f: funiculus; n: nucellus; mp: micropyle. Scale bar in **a** represents 250 μm in **a**, **b**; 50 μm in **c**, **f**; 40 μm in **d**, **g**, **k**; 125 μm in **e**; 100 μm in **h**; 80 μm in **i**, **j**

The similarities between the expression pattern and protein sequence of the tomato and Arabidopsis *INO* genes indicated that *SlINO* might play a role in tomato integument growth. Such a role would likely be conserved in closely related unitegmic solanaceous plants. As mutants of *INO* were not available in such species, virus induced gene silencing (VIGS) was used to engineer a knock-down of *INO* function in *N. benthamiana*. VIGS is especially effective in this species [30], including for the study of development [31]. *N. benthamiana* ovules [17] are very similar to those of tomato ([15, 16], and compare Fig. 2f, j, k to Fig. 3c, d, i) and VIGS is well established in this species. A genomic fragment containing most of the coding region of *NbINO1* (1162 bp) was used in a *Tobacco Rattle Virus* (TRV) silencing system [21]. Both *NbINO1* and *NbINO2* mRNA can be targeted for suppression by a single construct based on their high level of sequence identity (cDNAs are 97 % identical, Additional file 5: Figure S4a), and because the genomic clone used includes regions of perfect identity of 64, 65, 73, 85 and 148 bp between the two genes (Additional file 5: Figure S4b), and only 23 to 33 bp of identity are required for effective VIGS [32, 33]. From three separate experiments, a total of eleven plants treated with the silencing construct and eight control plants treated with the empty vector were compared 4–8 weeks after co-infiltration with *Agrobacteria* containing the constructs and the pTRV1 helper vector. Two to five carpels from each plant were examined with SEM and/or light microscopy (Fig. 3). The silencing system was shown to be functional by control infiltrations of a VIGS plasmid designed to silence *phytoene desaturase* and in all three experiments all such plants (*n* = 2, 2 and 1) produced the expected white leaves [21].

Development of the tobacco ovule is essentially identical to that described for tomato. In early stages of development, in wild type plants, the integument was first visible as a thickening on one side of the chalazal region of an ovule primordium (Fig. 3c). When the leading edge of the growing integument became clearly defined the ovule bent and the tip of the nucellus was directed toward the placental wall (Fig. 3d). During this time the ovules can be at various stages of development within a carpel. After further growth of the integument, the nucellus was fully enclosed, the ovules increase in diameter and mature wildtype ovules at anthesis appeared as tightly packed rounded structures (Fig. 3a, e). At this stage, the micropyle was not visible as it was tucked underneath the ovule due to asymmetric growth of the integument (Fig. 3a, e, i).

In VIGS knock-down plants, early growth of the integument appeared similar to that in control plants (Fig. 3c and f), but subsequently some ovules showed reduced curvature (Fig. 3g). In knock-down plants at anthesis, most ovules (50–100 % of ovules from nine carpels examined using SEM) differed from wild type, appearing elongated or oval in shape (Fig. 3b), not symmetrically round as in untreated plants (Fig. 3a). Severely aberrant ovules (Fig. 3b, h) with exposed nucelli resulting from a shortened integument were observed from every treated plant, with numbers ranging from 1 to 27 severely aberrant ovules in a single carpel (35 carpels examined from eleven plants, Additional file 6: Table S2). The total number of ovules in one carpel ranged from 100 to 140. Up to 20 % of ovules were severely aberrant, depending on the plant and experiment (Fig. 3b, h). The variable effects on ovules were likely due to variability in the efficiency of the VIGS in different plants, experiments, and locations on the placenta, but such ovules were never observed in non-silenced control plants treated with the empty vector (17 carpels from 8 plants). These severely affected ovules were not fully recurved and were sometimes nearly linear, and the nucellus was exposed due to incomplete development of the integument (Fig. 3h, j). The incomplete integument comprised two regions, a rounded basal region that had large cells in the outermost layer of the integument (Fig. 3h, j), similar to the cell layer covering the entire control ovules (Fig. 3i), and a thinner distal region with smaller evenly shaped cells. The ovules with the most aberrant overall morphology were those with the most reduced region of large surface cells (Fig. 3j).

The most severely affected ovules closely resemble ovules of the Arabidopsis double mutant *aberrant testa shape* (*ats*) *ino* (Fig. 3k). In *ats* mutants the two integuments are congenitally fused into a single integument [34] more closely resembling solanaceous ovules than those of wild-type Arabidopsis. The *ats ino* double mutant shows the effect of Arabidopsis *ino* on a unitegmic ovule that derives from two fused integuments: reduced growth of the single integument, and growth comprising two clear regions (Fig. 3k) as seen in *NbINO* knock-down tobacco. Thus the failure in integument extension in the ovules of *NbINO* knock-down tobacco plants and the similarity between these ovules and those of the Arabidopsis double mutant indicate similar roles for *INO* in tobacco and Arabidopsis ovules.

### Interspecies transfer shows incomplete conservation of promoter and protein function

*INO* gene expression and role in Solanaceae appears largely conserved with the *INO* gene in Arabidopsis and other studied plants. Such conservation of function may imply that the proteins and promoters have conserved specificities that can be tested by using heterologous transfer and observing the expression patterns and ability to complement the *ino* mutant.

A sequence comparison of either P-*SlINO* or a putative P*-NbINO* (2 kb of sequence upstream of translation start site) with 5’ sequence from Arabidopsis (P-*AtINO*) showed only very limited similarity that was confined to two regions previously defined as important for *AtINO* regulation, POS9 and POS6 [5, 35]. P-*SlINO* showed significant sequence conservation with P-*NbINO* in only one region, around −400 bp to −650 bp using both LAGAN [36] and EARS [37] analyses (Additional file 7: Figure S5).

**Fig. 4:**
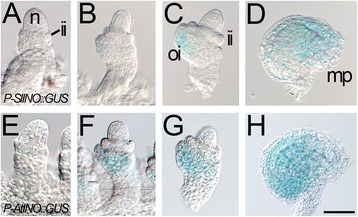
Conservation of *INO* promoter shown by activity of P-*SlINO* in Arabidopsis (L*er*), assayed using the GUS reporter. P-*SlINO::GUS* transgene activity (**d**-**g**) compared with P-*AtINO::GUS* transgene activity in (**e**-**h**). Early ovules (**a**, **e**) show no GUS activity in the emerging outer integument. Expression of the reporter in the outer integument is visible in ovules at stage 2-IV [63] but P-*SlINO* expression (**b**) is less extensive than P-*AtINO* at the same stage (**f**). This trend continues as the integuments extend towards the nucellus at stage 3-I (**c**, **g**). Finally, when the mature shape of the ovule is achieved at stage 4-I the pattern of GUS activity in **d** and **h** are similar though expression directed by P-*AtINO* (**h**) is concentrated toward the basal end of the integument as previously noted [5] while expression directed by P-*SlINO* is concentrated more toward the distal end of the integument. o: ovule; p: placenta; mp: micropyle; c: carpel wall; n: nucellus; ii: inner integument; oi: outer integument. Scale bar in **h** represents 25 μm in **a**-**c**, **e**-**g**; 50 μm in **d**, **h**

As a test of functional conservation of the promoters, P-*SlINO* was used to drive expression of ß-glucuronidase (GUS) in Arabidopsis (Fig. 4). The ovule-specific expression of *P-AtINO*:GUS in Arabidopsis has been well characterized [5, 35], starting with visible expression at the site of outer integument initiation and maintenance in the outer integument during growth, though expression is concentrated at the base of the integument, rather than the growing tip (Fig. 4e-h). When P-*SlINO*:GUS was analyzed in Arabidopsis, expression was also ovule-specific and observed only in the outer integument (10 transformants analyzed). The P-*SlINO* and P-*AtINO* plants were stained simultaneously, and minor but consistent differences in pattern were observed. P-*SlINO*:GUS ovules showed lighter staining of the outer integument early in development (Fig. 4b, f) and, at maturity, staining was more concentrated at the distal tip, unlike the pattern observed for P-*AtINO*:GUS (compare Fig. 4d, h).

**Fig. 5:**
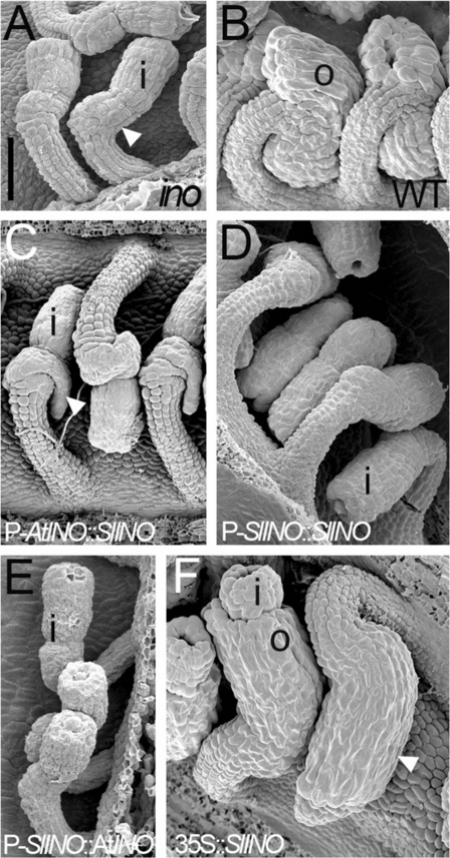
Transgenic complementation experiments of Arabidopsis *ino-1* by *SlINO* and *AtINO* expressed by P-*AtINO*, P-*SlINO* and the constitutive CaMV 35S promoter (all ovules at stage 4-I). *ino-1* ovules (**a**) show the effect of loss of *AtINO* on outer integument growth. The inner integument is intact and where the outer integument should have grown to cover it, as in the wildtype (**b**), no integument was initiated on the gynobasal side (arrowhead). The SlINO protein, when expressed using P-*AtINO* (**c**), cannot complement *ino-1* but leads to a small amount of misdirected growth (arrowhead) from the area where the outer integument should have arisen. This growth does not occur when P-*SlINO* is used to drive expression of *SlINO* (**d**) or *AtINO* (**e**). *SlINO* acts in a manner similar to other Arabidopsis YABBY genes when expressed using the constitutively active CaMV 35S promoter [64], promoting symmetrical growth of the outer integument (arrowhead) (**f**). This leads to a *sup-*like phenotype [5] in an *ino-1* mutant background, showing that the SlINO protein is functional and can effect outer integument growth. i: inner integument; o: outer integument; WT: wildtype. Scale bar in **a** represents 50 μm in panels **a**-**e** and 250 μm in **f**

As a further test of functional conservation of the Arabidopsis and tomato promoter regions, P-*SlINO* was used to express the Arabidopsis *AtINO* coding region in an *ino-1* mutant background to test for complementation of the mutant phenotype. Despite the expression pattern observed above, P-*SlINO:AtINO* was not able to complement the *ino* mutation, as outer integument growth was not restored in 6/6 *ino-1* primary transformants (Fig. 5e). This result shows that even though there is a similar expression pattern from both promoters, there are critical aspects of the promoters that are not conserved.

To evaluate conservation of protein function, SlINO was produced in Arabidopsis under the control of P-*AtINO*, to assay the protein’s ability to complement the *ino* mutant. In contrast to constructs with P-*AtINO* and the Arabidopsis *AtINO* coding region where a high frequency of full complementation was observed [35], the *SlINO* coding region was not effective at complementation. Four of six transgenic plants did not differ visibly from *ino-1* mutants, while the remaining two plants showed minimal, aberrant outer integument growth (Fig. 5c). P-*SlINO*::*SlINO* was also not able to complement the *ino-1* mutant (7/7 *ino-1* primary transfomants Fig. 5d)*.*

The failure of SlINO to complement *ino-1* may indicate divergence in protein structure that leads to failure to act on downstream targets required for integument growth. As it has been shown that *AtINO* participates in auto-regulation leading to continued expression after initiation of expression [5], one such target may be P-*AtINO* itself, with SlINO unable to maintain expression from P-*AtINO*. As a test of whether SlINO can function to support outer integument growth when expressed without regulatory constraints, a 35S::*SlINO* transgene was introduced into Arabidopsis (L*er*) segregating for the *ino-1* mutation. At least 25 % of 42 transformants showed phenotypes commonly observed with ectopic overexpression of any active YABBY gene, such as curled leaves and carpels [38] (data not shown). The CaMV 35S promoter is not reliably expressed in ovules, but in 16 % of the 42 independent transformants (all among those showing the vegetative phenotypic effects) there was evidence that SlINO was acting to promote outer integument growth. With the transgene in wildtype or heterozygous *INO/ino* backgrounds, symmetric growth of the outer integument was sometimes observed as compared with the normal asymmetric growth (3 plants). In four *ino-1* transgenic plants, outer integument growth was variable but substantially more than usually observed in the mutant and more symmetrical than in wild-type plants (Fig. 5f). Taken together, these overexpression results indicate that SlINO is a functional YABBY protein that retains at least some ability to promote outer integument growth in Arabidopsis.

## Discussion

The presence of two integuments is ancestral in the angiosperms [4] and the role of *INO* in integument development is conserved in examined bitegmic lineages in phylogenetically diverged clades [6, 7]. The unitegmic ovules of solanaceous species represent an evolutionary innovation that is common to all euasterids [1]. The expression pattern of *INO* orthologs in the outer layer of integumentary cells has been shown to be conserved in all previously examined unitegmic species such as *Prunus* [28] and *Impatiens* [11], but a role for this gene in such ovules has not been revealed through loss or reduction of function until this study.

The expression of *INO* in tomato is in only the outer integumentary layer preferentially on the side of the ovule with the greatest integument growth. This largely duplicates the pattern seen in all other angiosperms examined to date, including other members of the broader asterid clade (*Impatiens*) and members of the earliest branching angiosperm groups (Nymphaeales and Magnoliales). Hence it is reasonable to conclude that this pattern will also be in common with other members of the solanales with similar ovules such as tobacco. Indeed the effects of knock-down of *NbINO* in tobacco directly affect this layer, leading to a dramatic alteration in integument growth. The most affected ovules in the knock-down plants do not resemble those of loss of *INO* function in Arabidopsis or *Annona* (the only plants where such mutants have been examined), where the outer integument is completely absent and the inner integument grows normally and covers the nucellus. Instead the tobacco ovules appear to have reduced growth of the outermost portion of the single integument with continued proliferation of the interior material as an amorphous structure (Fig. 3h,j). While not resembling the Arabidopsis *ino* single mutant, the severely affected tobacco ovules are very similar in appearance to ovules of the Arabidopsis *ino ats* double mutant (Fig. 3k) [34]. The *ats* mutation causes congenital integument fusion, and the resulting unitegmic ovules in Arabidopsis resemble those of normal solanaceous plants [34]. The aberrant integument of both the tobacco knock-down and *ino ats* mutant ovules exhibit two surface cell types with small distal cells and larger cells on the surface of the proximal region. The origin of the distal region in the *ino ats* mutant is the inner layers of the single integument [34], and in tobacco the distal region derives from inner integumentary cell layers now exposed by reduction of the outer layer of enlarged cells. The extent of integument growth and resulting lack of ovule curvature in the knock-down plants correlated with the extent of the enlarged cells on the surface of the integuments. These cells correspond to those in which *SlINO* is expressed in tomato (and all other examined angiosperms), and the variability in growth of this region likely relates to variability in the effectiveness of gene suppression. Thus, in both tobacco and in Arabidopsis *ats ino* mutants, the loss or reduction in INO function leads to reduced outer cell layer proliferation and the resulting reduced and aberrant integumentary structures. The similarity of the effects of *ino* mutations on these two classes of unitegmic ovules indicates that the developmental role of *INO* is conserved between Arabidopsis and Solanales. Further, the most parsimonious explanation of the striking similarity of ovules produced in these two cases is that the single integument in Solanales evolved by congenital fusion of the ancestral inner and outer integuments (as produced by the *ats* mutant in Arabidopsis). Notably, one feature of *Impatiens* ovules that helped to identify unitegmic ovules as fused structures in that genus was retention of the differentiated endothelium as the inner layer of the single integument that corresponded to an anatomically similar layer in the inner integuments of bitegmic species [11]. The inner layer of ovules of the Solanaceae, such as tomato, also differentiates into an obvious endothelium [39]. Having an outer layer expressing the *SlINO* gene, and an inner layer differentiating into an endothelium, therefore, further supports the contention that the solanaceous integument is a fusion of ancestral outer and inner integuments.

Integument fusion was the cause of unitegmy in *Impatiens* [11] and, in a unitegmic *Prunus* species, reduced ATS and lack of production of ETTIN*,* an interaction partner of ATS, have been suggested as causing the evolution of unitegmy by fusion [28]*.* A similar mechanism may have been involved in the derivation of unitegmic ovules in the euasterids. Examination of this hypothesis could be the subject of future work.

Combining the results of species examined to date, we suggest that the role of *INO* in most species is to promote growth of the outer cell layer of a single or outer integument. This growth supports proliferation and organization of other attached layers to form an appropriately shaped laminar structure. Asymmetric distribution of *INO* expression results in asymmetric growth and the recurved, anatropous form of the ovules. In Arabidopsis, the spatial pattern of *AtINO* expression is controlled by *SUPERMAN* (*SUP*), which confines expression of *AtINO* to one side of the ovule by interfering with autoregulation [5]. The activity of SUP in the ovule appears to be confined to regulation of *AtINO* as *ino* mutations are fully epistatic to *sup* in the ovule [5]. This pathway appears to be conserved in the unitegmic ovules of *Petunia hybrida,* another Solanaceous species, where a mutation in *SUP* produces a phenotype similar to the Arabidopsis *sup* mutant – relatively symmetrical growth of the integument on all sides of the ovule [40]. This implies that the pathway controlled by SUP is conserved in the Solanaceae. Since SUP acts through regulation of *INO*, this provides further evidence for conservation of the role of *INO* in promotion of integument growth in the Solanaceae.

A conserved role is often expected to be associated with a conserved protein function. However, our results indicate that despite clear orthology and conserved developmental roles, tomato and Arabidopsis INO proteins are not interchangeable as SlINO could not complement an Arabidopsis *ino* mutant when expressed from P-*AtINO*. This is in contrast to similar experiments with other paralogous Arabidopsis YABBY proteins, which promote varying levels of outer integument growth [38, 41] despite lower levels of amino acid identity with AtINO (30 % to 35 %) than SlINO shares with AtINO (43 %). This result suggests that there are specific residues required for proper function of AtINO which are missing in SlINO, consistent with prior data that showed important determinants of AtINO function are distributed throughout the protein, rather than in a single structural domain [38, 41]. Taken together our results show that failure to complement in a heterologous expression experiment is not a reliable indicator that the proteins have diverged in their biological roles in the plants from which they derive.

In Arabidopsis, a functional AtINO protein is required for continued expression of P-*AtINO*, indicating autoregulation [5]. Thus, it is possible that the failure of SlINO to complement is due to either failure to activate P-*AtINO*, or failure to affect other downstream targets. We find that, when expressed under the CaMV 35S promoter, SlINO can promote integument growth indicating that it is capable of properly affecting downstream targets. Thus it could be that the major failure of SlINO in Arabidopsis is an inability to autoregulate through P-*AtINO*. However, it is also possible that SlINO has a reduced activity and overexpression from the 35S promoter can overcome this and produce sufficient activity to produce the necessary downstream effects. The current results do not allow differentiation of these two hypotheses.

Developmental regulators, including *INO,* are commonly expressed in spatially and temporally restricted zones, often mediated by strict transcriptional regulation. The domain of expression of *INO* in Arabidopsis and tomato is very similar with slightly different timing relative to initiation of the integument. The putative promoter of *SlINO* shares very little sequence identity with P-*AtINO* which is not unexpected due to the relative lack of conservation of promoter sequences. Remarkably, P-*SlINO* is able to produce in Arabidopsis a pattern of expression that is largely the same as that of the endogenous promoter suggesting that the native regulatory factors must be able to act on the non-native promoters despite their apparent lack of visible similarity. The slight differences observed in Arabidopsis – somewhat later initiation of expression, and expression tending more toward the distal end of the integument – echo the pattern of expression of the endogenous gene in tomato. This indicates that some of the differences in expression between these species derive from differences in the promoter sequences rather than only from differential presence of regulatory factors. Despite the similar expression pattern, P-*SlINO::AtINO* was not able to complement the *ino-1* mutation, perhaps due to late initiation of expression, or due to failure to autoregulate as has been shown to be necessary for P-*AtINO* [5]. Alternatively, while the 5’-region of the Arabidopsis *INO* gene has been shown to be a fully functional promoter [35] it is possible that the tomato promoter relies on distal elements that are missing from the tomato genomic fragment used.

Of all the YABBY family members *INO* seems most conserved in function across angiosperms. In all species studied, except those containing very recent duplications (e. g. *Nicotiana benthamiana*), there is only a single *INO* gene, and loss of function phenotypes of *Annona*, tobacco and Arabidopsis point to a conserved role. In contrast, there is evidence that the role of other YABBY genes may have diverged significantly. In general, the *FIL*, *YAB2* and *YAB5* sub-families are expressed and function in laminar leaf growth while the *CRC* and *INO* families play roles in reproductive structures, carpels and ovules, respectively [8, 42]. However, orthologs of *CRC* are expressed and function in leaf vasculature (*DROOPING LEAF* in rice, [43]), and nectary development (*CRC*, [44]). Gains of function in these cases may be related to changes in both expression and protein function. Similarly, an ortholog of *YAB2,* the *FASCIATED* gene of tomato, is an important determinant of fruit shape, affecting carpel number [22, 45] and is expressed in carpels, placenta and stamens as well as ovules. The apparently stronger evolutionary constraint on *INO*’s role may result from its importance in shaping integument architecture, which is crucial for efficient pollination and formation of the seed coat.

## Conclusions

Our results show that *INO* orthologs in the Solanaceae are essential to the growth and expansion of the outer layer(s) of ovule integuments, and hence the ancestral role of *INO* is largely conserved in the derived unitegmic ovules of this group. The results further indicate that the unitegmic ovules of tobacco (and hence other euasterid species) are most likely the result of a congenital fusion of two ancestral integuments. Thus, integument fusion produced unitegmy in the euasterids as was found for *Impatiens* in the closely related Ericales. This may indicate that the common ancestor to these groups was already genetically predisposed to this evolutionary pathway. The tomato *INO* ortholog was ineffective at complementing the Arabidopsis *ino* mutant, despite being more conserved in sequence than are other paralogous YABBY gene sequences that were effective at complementation. This indicates that specific residues along the protein are critical to species-specific function. In addition, it shows that cross-species complementation is not always an effective means to evaluate the conservation of the functional role of orthologous genes in different species.

## Methods

### Plant material

*Solanum lycopersicum* varieties Micro Tom (for in situ hybridizations) and Moneymaker (all transgenic work) and *Nicotiana benthamiana* were obtained from the Ralph M. Parsons Plant Transformation facility at U. C. Davis. *S. lycopersicum* variety VF36 was obtained from the Tomato Genetics Resource Center at U. C. Davis. Arabidopsis (Landsberg *erecta*) *ino-1* mutant was previously published [6].

### DNA constructs

A tomato *INO* (*SlINO*) cDNA clone was isolated from a tomato pistil cDNA library constructed in λ-GT10 from strain VF36 [25] using the Arabidopsis *AtINO* cDNA [6] as a hybridization probe. The sequence of this clone differs by a single base from the cDNA predicted from gene Solyc05g005240.1.1 in the reference tomato genome [20]. The base substitution produces a conservative substitution of a valine in the VF36 cDNA for a corresponding isoleucine in the reference sequence. The tomato cDNA was used to screen a tomato genomic library [26] to isolate a genomic clone that included the 2257 bp of 5’ flanking sequence extending up to the next putative gene (Solyc05g005250.2.1, pantothenate kinase). The clone also included four *SlINO* exons that confirmed that the single base change observed in the cDNA was a natural polymorphism between VF36 and the reference genome.

The *SlINO* cDNA coding region was inserted between the promoter and 3′ flanking regions of *AtINO* as previously described [35] to create a P-*AtINO::SlINO::AtINO3′* transgene (pRKK170). The 2257 promoter region of *SlINO* (P-*SlINO*) was combined with the *SlINO* cDNA as a translational fusion with the GREEN FLUORESCENT PROTEIN (pGFP1.1.5, [46]) and the nopaline synthase 3′-end (Nos3′) to make P-*SlINO::SlINO:GFP::NOS3′* (pRB30). P-*SlINO* was inserted into pHK17 [47] to drive expression of *E. coli* ß-glucuronidase (GUS, [48]) in a P-*SlINO::GUS::NOS3′* transgene (pRB8). P-*SlINO* was combined with both the *AtINO* cDNA and Nos3′ to make P-*SlNO::AtINO::NOS3′* (pRB67(71)) and with *SlINO* cDNA and Nos3′ to make P-*SlINO::SlINO::NOS3′* (pRB66(68)) which were used to assess complementation in Arabidopsis.

### Plant transformation

For plant transformation, chimeric transgenes were transferred as NotI fragments into pART27 [49] or pMLBART [49], which confer kanamycin or phosphinothricine resistance on transformed tomato or Arabidopsis plants, respectively. Transformations of the tomato Moneymaker variety were performed with *Agrobacterium* LBA4404 [50] at the Ralph M. Parsons plant transformation facility by published methods [51]. Transformation of Arabidopsis (Landsberg *erecta*) utilized either *Agrobacterium* LBA4404 or ASE [52] and the floral dip method [53].

### Virus induced gene silencing

Genomic *NbINO* was amplified using Phusion Hifi taq polymerase with primers F1 atctagaATGTCAGCATTGAATCATCTGTTTG and R1 actcgagCTTTGGCATCTTTCTGTCTCC adding Xba1 and Xho1 sites. The product was blunt cloned into pJET1.2 (ThermoFisher, Waltham, MA) according to manufacturer’s directions to make pDS207, which was sequenced to verify insert integrity. The insert was transferred using Xba1/Xho1 digestion and ligation into pYL156, the TRV2 vector [54, 55], to make pDS208. pDS208 was transferred into Agrobacterium GV2260 and used for VIGS via infiltration of *N. benthamiana* leaves [21] in three separate experiments. A control VIGS experiment using *PHYTOENE DESATURASE* (*NbPDS*) showed white leaves by 2 weeks after infiltration. Ovules from *NbINO* silenced plants were examined with SEM from 4 weeks after infiltration and compared to plants infiltrated with an empty silencing vector.

### Microscopy

GUS staining followed the methods of Meister et al. [5]. Stained and unstained ovules were visualized under differential interference contrast (DIC) using a Zeiss Axioplan 2 microscope. Confocal images of tomato ovules expressing SlINO-GFP fusion protein were taken with a Zeiss LSM 710 system using 488 nm laser excitation and recording 493–598 nm emission wavelengths (Carl Zeiss Inc, Germany). For scanning electron micrography (SEM), Arabidopsis tissue was fixed and critical point dried as previously described [11], and tobacco carpels were treated similarly but each fixation step was twice as long, and three additional five minute soaks in liquid CO_2_ were used during critical point drying. Samples were imaged on a Philips XL 30 SEM (FEI Company, http://www.fei.com). In situ hybridizations on tomato (Microtom) followed the procedures described in Skinner and Gasser [56]. The hybridization probe derived from a 531 bp fragment isolated by PCR from the SlINO cDNA and containing the 3′ untranslated region and regions encoding the YABBY and central diverged domains. This fragment was inserted into pCRII-TOPO (Invitrogen/ThermoFisher, Carlsbad, CA) oriented with the 5′-end adjacent to the XhoI site. Digestion with XhoI and transcription with SP6 RNA polymerase (Promega, Madison, WI) produced the antisense hybridization probe.

## Abbreviations

ATS, Abberant testa shape; CaMV, cauliflower mosaic virus; cDNA, complementary DNA; CRC, CRABS CLAWS; DIC, differential interference contrast; FIL, filamentous flower; GUS, β-glucuronidase; GFP, green fluorescent protein; INO, inner no outer; SUP, superman; TRV, tobacco rattle virus; VIGS, virus induced gene silencing; YAB2/3/5, YABBY 2/3/5.

## Additional files

Additional file 1: Figure S1. Phylogenetic relationships of YABBY proteins from representative species. Amino acid sequences of YABBY proteins from representative species (all known YABBY proteins from *Arabidopsis thaliana* (At), *Cabomba caroliniana* (Cc), tomato (Sl), rice (Os), and *Amborella trichopoda* (Amb), and example sequences from *Antirrhinum majus* (Am), *Vitus vinerfa* (Vv), *Nymphaea alba* and *colorata* (Na and Nc) and *Pinus stichensis* (Ps, used as the root) were aligned using Clustal X v. 2.1 [57]. Phylogenetic relationships were evaluated for maximum parsimony in PAUP 4.0b10 [58] using a heuristic search and the branch and bound algorithm. Characters were weighted using a BLOSUM62 [59] matrix as described [11]. The illustrated phylogram is the single shortest tree which was found in more than 49 % of 1000 replicate searches. Branch lengths are proportional to number of changes in arbitrary units. Statistical significance of clades was evaluated with 1000 bootstrap resamplings using the same search criteria but with five replicate searches for each resampling. Bootstrap values ≥ 50 % are shown on the supported clades. Sources of tomato and tobacco sequences are in Additional file 2: Table S1 and Additional file 3: Figure S2, and Genbank accession numbers of the other sequences are listed at the end of each taxon. Labels at far right are the five cannonical clades of YABBY proteins, color coordinated with the associated clade. The CRC and INO clades have especially strong statistical support. (PDF 356 kb) Additional file 2: Table S1. Proposed *S. lycopersicon* tomato orthologs of Arabidopsis *YABBY* gene family members ([22], Fig. S1). (DOCX 52 kb) Additional file 3: Figure S2. CLUSTAL O (1.2.1) multiple sequence alignment of Arabidopsis INO, and orthologous proteins from *S. lycopersicon* (Solyc05g005240, SlINO) and *N. benthamiana* (our predicted version of Niben101Scf09599g00012.1, NbINO1 and Niben101Scf04287g04009.1, NbINO2). (DOCX 114 kb) Additional file 4: Figure S3. The P-*SlINO*::*SlINO*-*GFP* transgene continues to be expressed after fertilization during the onset of fruit development. **A**-**C**: Ovules from P-*SlINO*::*SlINO*-*GFP* plants. **D**, **E**: Ovules from control plants. Images **A** (confocal) and **B** (DIC overlaid with GFP channel) show expression in the outer cell layer in an ovule post-anthesis. **C**-**E** are images of the surface cells of the integument of ovules taken from 3–4 mm fruits. **C** and **D** are images taken on an epifluorescence microscope (Axioplan) using a Chroma GFP filter set 41017 (Chroma, Bellows Falls, VT). **E** is a dark-field image of the same ovule in **D**. These images show expression is present in developing fruit. Scale bar in **B** represents 20 μm, scale bar in **E** represents 20 μm in **C**-**E**. (TIF 4435 kb) Additional file 5: Figure S4. Alignment of the two N. Benthamiana *INO* sequences showing regions of identity. **A** shows alignment of the coding sequences of *NbINO1 and NbINO2* and **B** shows alignment of genomic fragment used for VIGS. Underlined regions are long stretches of identical sequences likely to be effective for VIGS and blue regions indicate exons. Alignment was performed using an implementation of the Needleman-Wunsch algorithm [60] as implemented at http://www.ebi.ac.uk/Tools/psa/emboss_needle/nucleotide.html. (DOCX 107 kb) Additional file 6: Table S2. Results of VIGS experiments showing the number of plants and carpels analyzed. (DOCX 64 kb) Additional file 7: Figure S5. Comparison of *INO* promoters. **A**, **B**, and **C** show results of EARS analysis [37]. X-axis represents *P-*values for maximum alignment score for a 60 bp window from each promoter to its best matching window in the Arabidopsis sequence (**A**,**B**) or tobacco (**C**). A *P*-value less than 10^−4^ indicates conservation. **D** shows the result of a multispecies comparison of promoters using the LAGAN method [36] and VISTA tools [61] (http://genome.lbl.gov/vista/mvista/submit.shtml). Promoters from tobacco, tomato and *Brassica oleracea* (P-*BoINO*) are compared with P-*AtINO* (y-axis). Pink regions visible in P-*BoINO* comparison indicate high conservation of the POS6, POS9 and Minimal Promoter regions [62] of which only a small part of POS9 and POS6 are conserved at a very low level in the promoters from tobacco and tomato. (PDF 340 kb)
