# Supplementary figures and images for: Conservation of the role of INNER NO OUTER in development of unitegmic ovules of the Solanaceae despite a divergence in protein function
Source: BMC Plant Biol. 2016 Jun 27;16:143. doi: 10.1186/s12870-016-0835-z (PMC4924249; doi:10.1186/s12870-016-0835-z)

Figure S1

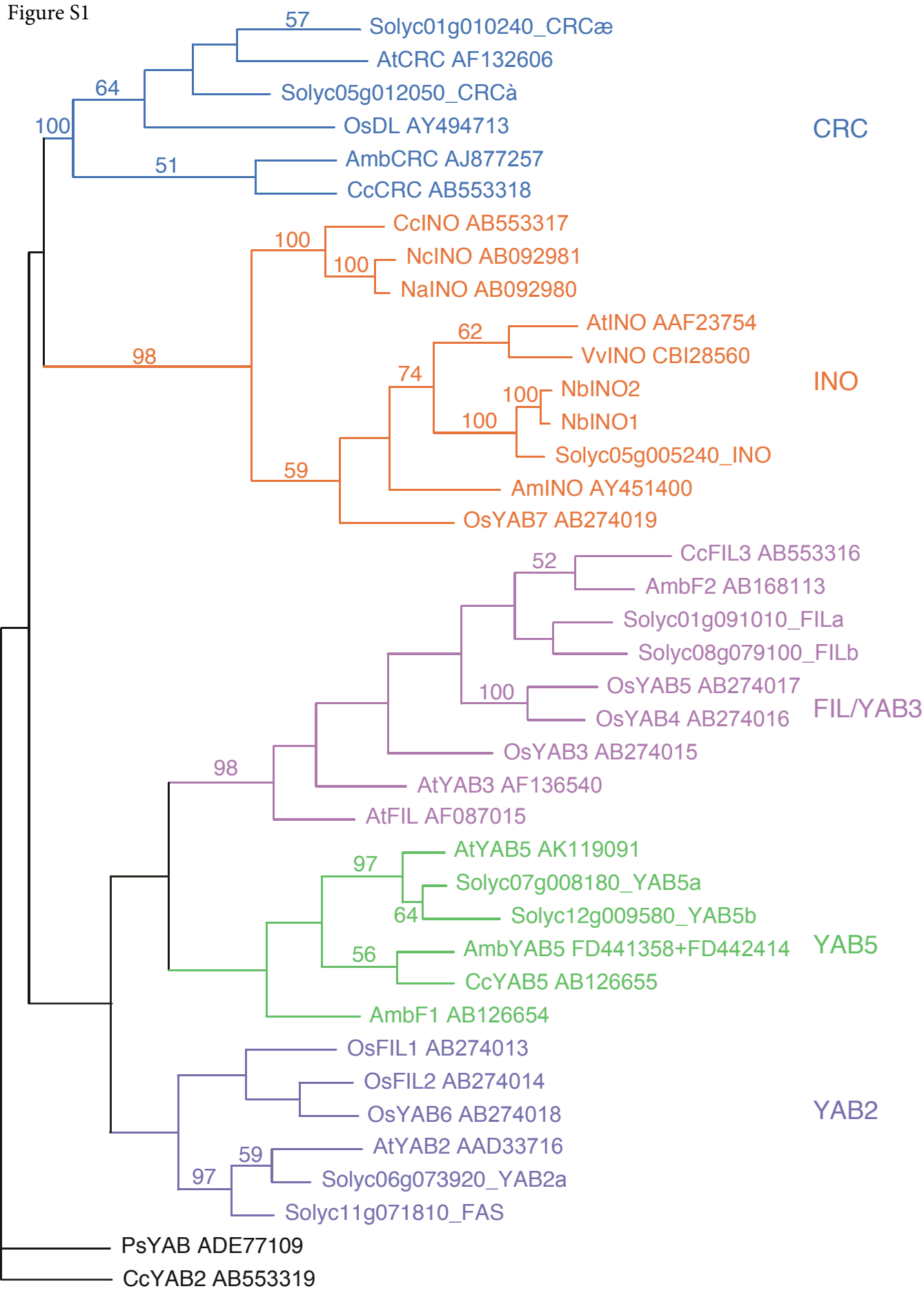

Supplement: Additional file 1: Figure S1. — Phylogenetic relationships of YABBY proteins from representative species. Amino acid sequences of YABBY proteins from representative species (all known YABBY proteins from Arabidopsis thaliana (At), Cabomba caroliniana (Cc), tomato (Sl), rice (Os), and Amborella trichopoda (Amb), and example sequences from Antirrhinum majus (Am), Vitus vinerfa (Vv), Nymphaea alba and colorata (Na and Nc) and Pinus stichensis (Ps, used as the root) were aligned using Clustal X v. 2.1 [57]. Phylogenetic relationships were evaluated for maximum parsimony in PAUP 4.0b10 [58] using a heuristic search and the branch and bound algorithm. Characters were weighted using a BLOSUM62 [59] matrix as described [11]. The illustrated phylogram is the single shortest tree which was found in more than 49 % of 1000 replicate searches. Branch lengths are proportional to number of changes in arbitrary units. Statistical significance of clades was evaluated with 1000 bootstrap resamplings using the same search criteria but with five replicate searches for each resampling. Bootstrap values ≥ 50 % are shown on the supported clades. Sources of tomato and tobacco sequences are in Additional file 2: Table S1 and Additional file 3: Figure S2, and Genbank accession numbers of the other sequences are listed at the end of each taxon. Labels at far right are the five cannonical clades of YABBY proteins, color coordinated with the associated clade. The CRC and INO clades have especially strong statistical support. (PDF 356 kb) [file 12870_2016_835_MOESM1_ESM.pdf]

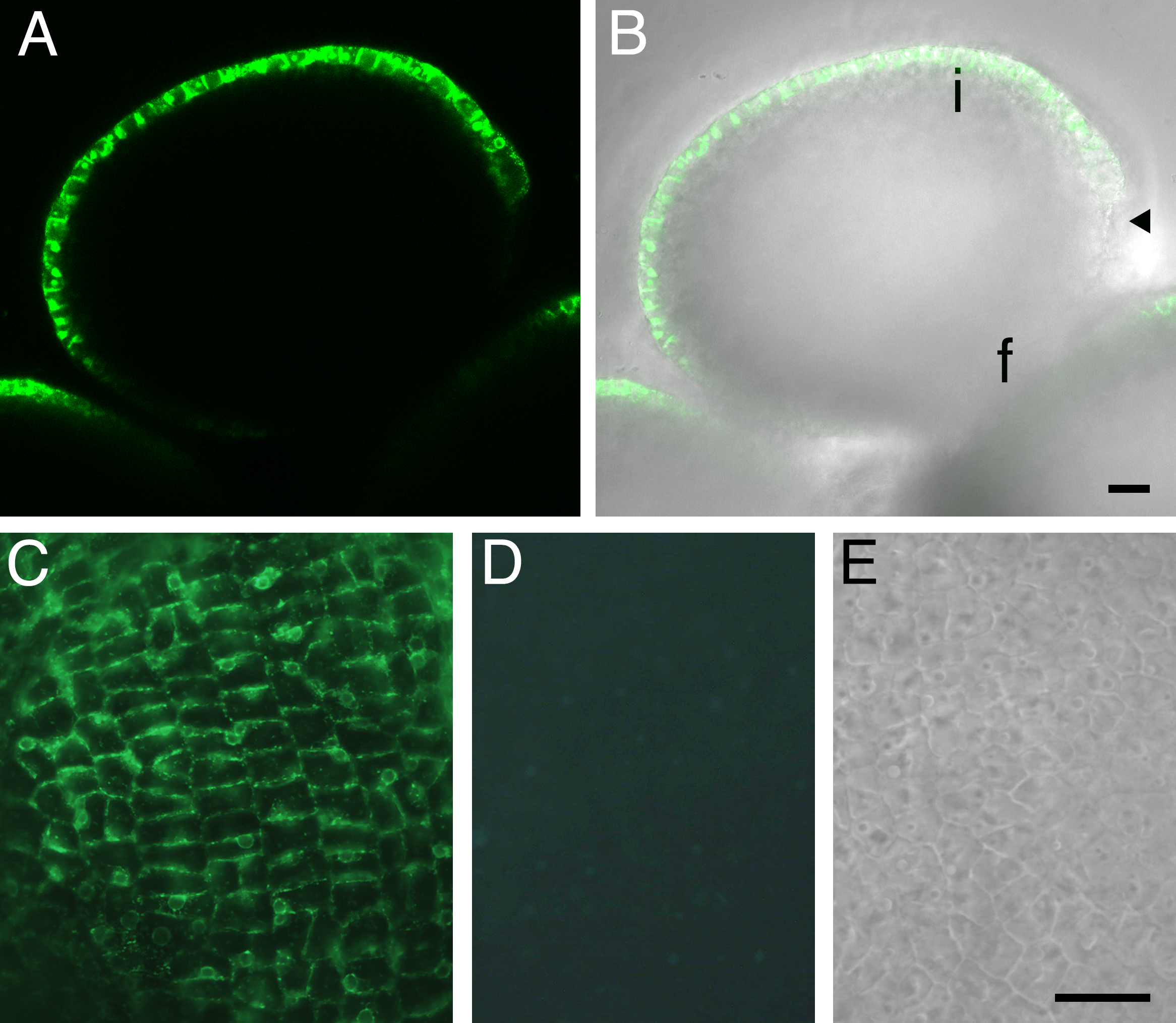

Supplement: Additional file 4: Figure S3. — The P-SlINO::SlINO-GFP transgene continues to be expressed after fertilization during the onset of fruit development. A-C: Ovules from P-SlINO::SlINO-GFP plants. D, E: Ovules from control plants. Images A (confocal) and B (DIC overlaid with GFP channel) show expression in the outer cell layer in an ovule post-anthesis. C-E are images of the surface cells of the integument of ovules taken from 3–4 mm fruits. C and D are images taken on an epifluorescence microscope (Axioplan) using a Chroma GFP filter set 41017 (Chroma, Bellows Falls, VT). E is a dark-field image of the same ovule in D. These images show expression is present in developing fruit. Scale bar in B represents 20 μm, scale bar in E represents 20 μm in C-E. (TIF 4435 kb) [file 12870_2016_835_MOESM4_ESM.tif]

Figure S5

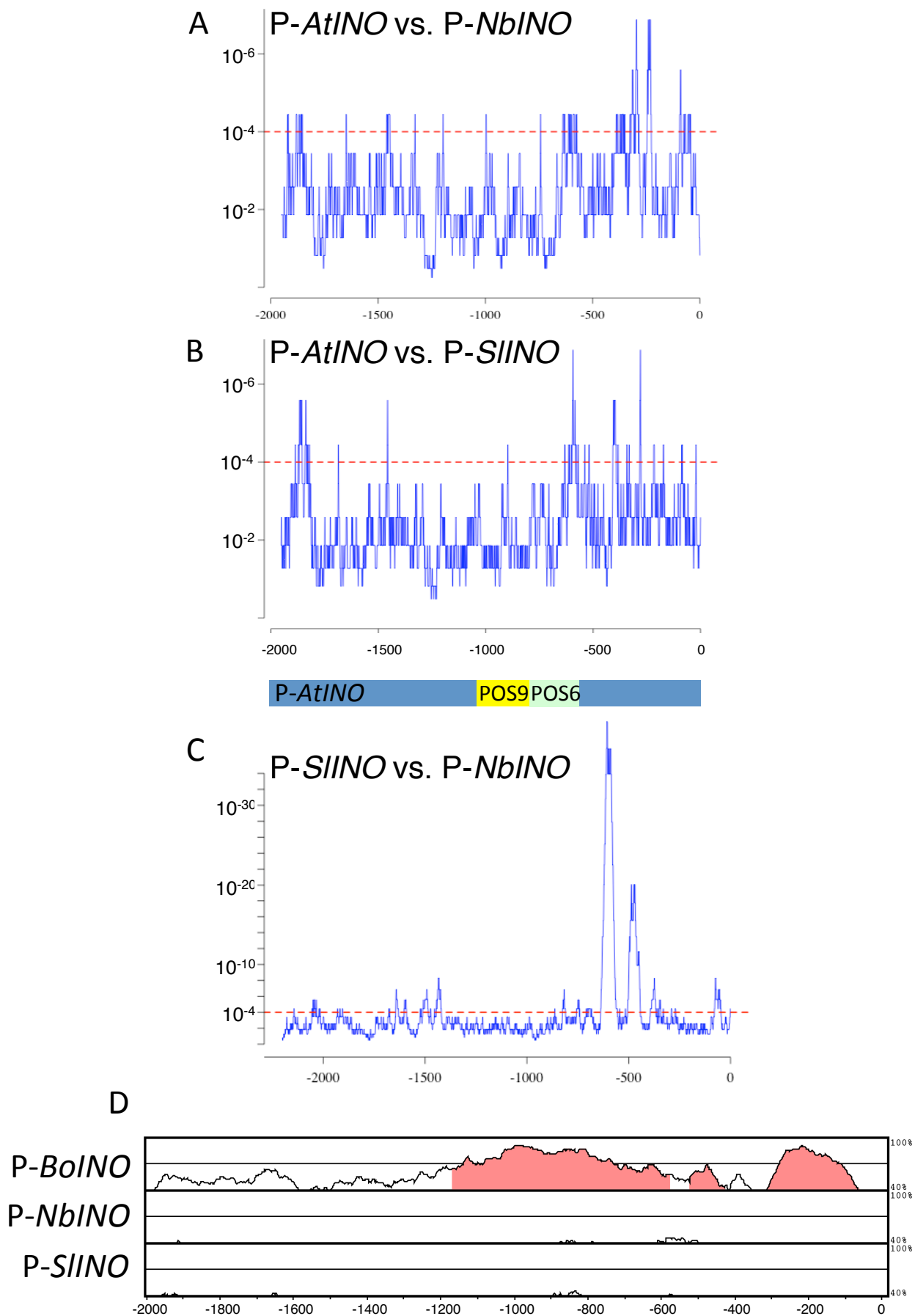

Supplement: Additional file 7: Figure S5. — Comparison of INO promoters. A, B, and C show results of EARS analysis [37]. X-axis represents P-values for maximum alignment score for a 60 bp window from each promoter to its best matching window in the Arabidopsis sequence (A,B) or tobacco (C). A P-value less than 10−4 indicates conservation. D shows the result of a multispecies comparison of promoters using the LAGAN method [36] and VISTA tools [61] (http://genome.lbl.gov/vista/mvista/submit.shtml). Promoters from tobacco, tomato and Brassica oleracea (P-BoINO) are compared with P-AtINO (y-axis). Pink regions visible in P-BoINO comparison indicate high conservation of the POS6, POS9 and Minimal Promoter regions [62] of which only a small part of POS9 and POS6 are conserved at a very low level in the promoters from tobacco and tomato. (PDF 340 kb) [file 12870_2016_835_MOESM7_ESM.pdf]
