# Supplementary material for: Conservation of the role of INNER NO OUTER in development of unitegmic ovules of the Solanaceae despite a divergence in protein function
Source: BMC Plant Biol. 2016 Jun 27;16:143. doi: 10.1186/s12870-016-0835-z (PMC4924249; doi:10.1186/s12870-016-0835-z)
Supplement: Additional file 2: Table S1. — Proposed S. lycopersicon tomato orthologs of Arabidopsis YABBY gene family members ([22], Fig. S1). (DOCX 52 kb) [file 12870_2016_835_MOESM2_ESM.docx]

**Table S1.** Proposed *S. lycopersicon* tomato orthologs of Arabidopsis *YABBY* gene family members [22], our analysis.

| **Gene locus** | **Gene name** | **Arabidopsis Gene** |
| --- | --- | --- |
| Solyc5g005240 | *SlINO* | *INO* |
| Solyc1g010240 | *SlCRCa* | *CRC* |
| Solyc5g012050 | *SlCRCa* | *CRC* |
| Solyc1g091010 | *SlYABBY1a* | *FIL/YAB3* |
| Solyc8g079100 | *SlYABBY1b* | *FIL/YAB3* |
| Solyc6g073920 | *SlYABBY2a* | *YAB2* |
| Solyc11g071810 | *FAS* | *YAB2* |
| Solyc7g008180 | *SlYABBY5a* | *YAB5* |
| Solyc12g009580 | *SlYABBY5b* | *YAB5* |
