# Supplementary material for: Conservation of the role of INNER NO OUTER in development of unitegmic ovules of the Solanaceae despite a divergence in protein function
Source: BMC Plant Biol. 2016 Jun 27;16:143. doi: 10.1186/s12870-016-0835-z (PMC4924249; doi:10.1186/s12870-016-0835-z)
Supplement: Additional file 3: Figure S2. — CLUSTAL O (1.2.1) multiple sequence alignment of Arabidopsis INO, and orthologous proteins from S. lycopersicon (Solyc05g005240, SlINO) and N. benthamiana (our predicted version of Niben101Scf09599g00012.1, NbINO1 and Niben101Scf04287g04009.1, NbINO2). (DOCX 114 kb) [file 12870_2016_835_MOESM3_ESM.docx]

**Figure S2.** CLUSTAL O (1.2.1) multiple sequence alignment of AtINO, and homologous proteins from *S. lycopersicon* (Solyc05g005240, SlINO) and *N. benthamiana* (our predicted version of Niben101Scf09599g00012.1, NbINO1 and Niben101Scf04287g04009.1, NbINO2).

AtINO MTKLPNMTTTLNHLFDLPGQICHVQCGFCTTILLVSVPFTSLS-MVVTVRCGHCTSLLSV 59

SlINO ------MSTLNNHLFELQDTICYVQCGYCTTILLVSVPCSSLCNKVVTVRCGHCTTLLSL 54

NbINO1 -------MSALNHLFELQDTICYVQCGYCTTILLVSVPCSSLCNKIVTVRCGHCTSLLSV 53

NbINO2 -------MSALNHLFELQDTICYVQCGYCTTILLVSVPCSSLCNKIVTVRCGHCTSILSV 53

: ****:* **:****:********** :**. :*********::**:

AtINO NLMKASFIPLHLLASLSHLDETGKEEVAATDGVEEEAWKVNQEKENSPTTLVSSSDNE-- 117

SlINO NLIKPS---LHLFASFDQTHQPP-E-VDKD-----ETDDANKKN--------SNSDEEDQ 96

NbINO1 NLMKASLVPLHLFASLNLTE-QKLE-VDKE-----DI-DANKKSVDSEISFVASSDEEDQ 105

NbINO2 YLMKTSLVPLHLFASLNQSEQQKLE-VDKE-----DI-DANKKSVDSEISFVASSDEEDQ 106

*:* * ***:**:. . * * : ..*::. :.**:*

AtINO -DEDVSRVYQVVNKPPEKRQRAPSAYNCFIKEEIRRLKAQNPSMAHKEAFSLAAKNWAHF 176

SlINO LENNVLPLNQVVNKPPEKRQRAPSAYNCFIKEEIKRLKTLYPNMTHKQAFSTAAKNWAHF 156

NbINO1 I-ENVVPVYQVVNKPPEKRQRAPSAYNCFIKEEIKRLKTIYPNMTHKQAFSTAAKNWAHF 164

NbINO2 I-ENVVPVYQVVNKPPEKRQRAPSAYNCFIKEEIKRLKTIYPNMTHKQAFSTAAKNWAHF 165

::* : *************************:***: *.*:**:*** ********

AtINO PPAHNKRAASDQCFC---------------EEDNNAILPCNVFEDHEESNNGFRERKAQR 221

SlINO PPSQHR---GGCSLGERKMAKVSAARNSMVPRDSNGLIP--------------------- 192

NbINO1 PPSQHREDRESCSLGDRKMPKASK------------------------------------ 189

NbINO2 PPSQHRGDRESCSLGDRKMPKVLS------------------------------------ 189

**::.: .:

AtINO HSIWGKSPFE 231

SlINO ---------- 192

NbINO1 ---------- 189

NbINO2 ---------- 189
