# Supplementary material for: Conservation of the role of INNER NO OUTER in development of unitegmic ovules of the Solanaceae despite a divergence in protein function
Source: BMC Plant Biol. 2016 Jun 27;16:143. doi: 10.1186/s12870-016-0835-z (PMC4924249; doi:10.1186/s12870-016-0835-z)
Supplement: Additional file 5: Figure S4. — Alignment of the two N. Benthamiana INO sequences showing regions of identity. A shows alignment of the coding sequences of NbINO1 and NbINO2 and B shows alignment of genomic fragment used for VIGS. Underlined regions are long stretches of identical sequences likely to be effective for VIGS and blue regions indicate exons. Alignment was performed using an implementation of the Needleman-Wunsch algorithm [60] as implemented at http://www.ebi.ac.uk/Tools/psa/emboss_needle/nucleotide.html. (DOCX 107 kb) [file 12870_2016_835_MOESM5_ESM.docx]

**Figure S4:** Alignment of two *INO* N. Benthamiana sequences using the Needleman-Wunsch algorithm.

**A: Coding sequences of *N. benthamiana* *INO1* and *INO2***

NbINO1 1 ATGTCAGCATTGAATCATCTGTTTGAACTCCAAGACACAATCTGTTACGT 50

||||||||||||||||||||||||||||||||||||||||||||||||||

NbINO2 1 ATGTCAGCATTGAATCATCTGTTTGAACTCCAAGACACAATCTGTTACGT 50

NbINO1 51 GCAATGTGGCTACTGCACTACAATATTACTTGTTAGTGTCCCATGCAGCA 100

||||||||||||||||||||||||||||||||||||||||||||||||||

NbINO2 51 GCAATGTGGCTACTGCACTACAATATTACTTGTTAGTGTCCCATGCAGCA 100

NbINO1 101 GCTTATGCAATAAGATAGTAACAGTGAGATGTGGCCATTGCACTAGCCTC 150

|||||||||||||||||||||||||||||||||||||||||||||||.||

NbINO2 101 GCTTATGCAATAAGATAGTAACAGTGAGATGTGGCCATTGCACTAGCATC 150

NbINO1 151 CTTTCTGTTAACTTGATGAAAGCATCTCTTGTTCCTCTTCATCTTTTTGC 200

|||||||||.|||||||||||.|.||||||||||||||||||||.|||||

NbINO2 151 CTTTCTGTTTACTTGATGAAAACTTCTCTTGTTCCTCTTCATCTCTTTGC 200

NbINO1 201 TTCCCTTAACCTAACTGAGCAGCAAAAGCTAGAAGTTGACAAGGAGGACA 250

|||||||||||.|.|.||||||||||||||||||||||||||||||||||

NbINO2 201 TTCCCTTAACCAATCCGAGCAGCAAAAGCTAGAAGTTGACAAGGAGGACA 250

NbINO1 251 TTGATGCTAATAAGAAGAGTGTAGACTCAGAAATCTCATTTGTGGCCTCA 300

||||||||||||||||||||||||||||||||||||||||||||||||||

NbINO2 251 TTGATGCTAATAAGAAGAGTGTAGACTCAGAAATCTCATTTGTGGCCTCA 300

NbINO1 301 TCAGATGAAGAAGATCAAATAGAGAACGTTGTTCCAGTTTATCAAGTCGT 350

||||||||||||||||||||||||||.|||||||||||||||||||||||

NbINO2 301 TCAGATGAAGAAGATCAAATAGAGAATGTTGTTCCAGTTTATCAAGTCGT 350

NbINO1 351 CAATAAACCTCCAGAGAAAAGACAACGAGCCCCATCAGCTTATAACTGCT 400

|||.||||||||||||||||||||||||||||||||||||||||||||||

NbINO2 351 CAACAAACCTCCAGAGAAAAGACAACGAGCCCCATCAGCTTATAACTGCT 400

NbINO1 401 TTATCAAAGAAGAGATCAAGAGGCTAAAGACCATATACCCCAACATGACT 450

||||||||||||||||||||||||||||||||||||||||||||||||||

NbINO2 401 TTATCAAAGAAGAGATCAAGAGGCTAAAGACCATATACCCCAACATGACT 450

NbINO1 451 CACAAGCAAGCTTTCAGTACGGCAGCAAAAAATTGGGCCCACTTTCCACC 500

|||||||||||.||||||||.|||||||||||||||||||||||||||||

NbINO2 451 CACAAGCAAGCCTTCAGTACCGCAGCAAAAAATTGGGCCCACTTTCCACC 500

NbINO1 501 AAGTCAGCATAGAGAAGATAGAGAAAGCTGTAGCCTGGGAGACAGAAAGA 550

||||||.|||||||.|||||||||||||||||||||.|||||||||||||

NbINO2 501 AAGTCAACATAGAGGAGATAGAGAAAGCTGTAGCCTAGGAGACAGAAAGA 550

NbINO1 551 TGCCAAAG 558

||||||||

NbINO2 551 TGCCAAAG 558

**B: Genomic region of *N*. *benthamiana* *INO1* and *INO2* used for VIGS.**

Identical region lengths: 85, 73, 148, 65, 64 (underlined)

NbINO1 1 ATGTCAGCATTGAATCATCTGTTTGAACTCCAAGACACAATCTGTTACGT 50

||||||||||||||||||||||||||||||||||||||||||||||||||

NbINO2 1 ATGTCAGCATTGAATCATCTGTTTGAACTCCAAGACACAATCTGTTACGT 50

NbINO1 51 GCAATGTGGCTACTGCACTACAATATTACTTGTAAAATTAC--ATGTCTT 98

|||||||||||||||||||||||||||||||||||.||||| ||.||||

NbINO2 51 GCAATGTGGCTACTGCACTACAATATTACTTGTAAGATTACATATATCTT 100

NbINO1 99 TTTTGCGTTT---TAGTTACTCTTTATCTCTATAT---TTTTATCGGTTA 142

|.||.| |.| ||..||| |||.||||||||.| ||||||||||||

NbINO2 101 TCTTTC-TCTCAATATATAC-CTTCATCTCTATGTATATTTTATCGGTTA 148

NbINO1 143 -----ATTTGTTATCCAAAA----TATTGCAGGTTAGTGTCCCATGCAGC 183

||||||||||||||| .|||||||||||||||||||||||||

NbINO2 149 ATTTTATTTGTTATCCAAAAAATTAATTGCAGGTTAGTGTCCCATGCAGC 198

NbINO1 184 AGCTTATGCAATAAGATAGTAACAGTGAGATGTGGCCATTGCACTAGCCT 233

||||||||||||||||||||||||||||||||||||||||||||||||.|

NbINO2 199 AGCTTATGCAATAAGATAGTAACAGTGAGATGTGGCCATTGCACTAGCAT 248

NbINO1 234 CCTTTCTGTTAACTTGATGAAAGCATCTCTTGTTCCTCTTCATCTTTTTG 283

||||||||||.|||||||||||.|.||||||||||||||||||||.||||

NbINO2 249 CCTTTCTGTTTACTTGATGAAAACTTCTCTTGTTCCTCTTCATCTCTTTG 298

NbINO1 284 CTTCCCTTAACCTAACTGAGGTCGAACTATAATCTTATTTAAATAATCTC 333

||||||||||||.|.|.|||||.||||.||..||.|||| |||||||

NbINO2 299 CTTCCCTTAACCAATCCGAGGTTGAACGATTGTCGTATT----TAATCTC 344

NbINO1 334 TATTGAGTTTAATTTCTATACATTAATCG----CATCATG-ACAAGAATA 378

|.||||||||||||||||||||||||||| |.||||| |.|||||..

NbINO2 345 TTTTGAGTTTAATTTCTATACATTAATCGTGATCGTCATGCAAAAGAAAG 394

NbINO1 379 ATTATTAATCTGAAAAATATATAAGTCATGTTACCTGTTATGAC----TT 424

||||.||||||||||| |.|| |.|||||.|| ||

NbINO2 395 ATTAGTAATCTGAAAA----------CCTG----CAGTTATAACTTAATT 430

NbINO1 425 ATTTAAA-AAATTAGA----------ATTTTTACACTGTCATT----ATA 459

||||||| |.||| || |||||||||.|||||.| |||

NbINO2 431 ATTTAAATACATT-GATATATAGTGCATTTTTACATTGTCACTCTACATA 479

NbINO1 460 TGTTAAATTAATTTTTTCCCGCTTTTCTCTCGATGTTAATTGACTTAGAA 509

||||||.||||||| ||||||.|||||||||||||||||||||

NbINO2 480 TGTTAACTTAATTT--------TTTTCTTTCGATGTTAATTGACTTAGAA 521

NbINO1 510 TCTATCTTTCCCGCTTTCAGCAGCAAAAGCTAGAAGTTGACAAGGAGGAC 559

||||||||||||||||||||||||||||||||||||||||||||||||||

NbINO2 522 TCTATCTTTCCCGCTTTCAGCAGCAAAAGCTAGAAGTTGACAAGGAGGAC 571

NbINO1 560 ATTGATGCTAATAAGAAGAGTGTAGACTCAGAAATCTCATTTGTGGCCTC 609

||||||||||||||||||||||||||||||||||||||||||||||||||

NbINO2 572 ATTGATGCTAATAAGAAGAGTGTAGACTCAGAAATCTCATTTGTGGCCTC 621

NbINO1 610 ATCAGATGAAGAAGATCAAATAGAGAACGTTGTTCCAGTTTATCAAGTCG 659

|||||||||||||||||||||||||||.||||||||||||||||||||||

NbINO2 622 ATCAGATGAAGAAGATCAAATAGAGAATGTTGTTCCAGTTTATCAAGTCG 671

NbINO1 660 TCAATAAACGTATACTATCTAAAAAGAAAAAAAATTTATATAGATGATTT 709

||||.|||||||.|.|||||| ||.||||||||||||

NbINO2 672 TCAACAAACGTAAAATATCTA--------------TTGTATAGATGATTT 707

NbINO1 710 AAACTGTCCTAATTAATTATTTTTACGTTACTCACA----ATAAGAATCT 755

|||.|||||||||||||||||||||||||||||||| ||||||||||

NbINO2 708 AAATTGTCCTAATTAATTATTTTTACGTTACTCACAATATATAAGAATCT 757

NbINO1 756 AATCAT----ATTTTTTCAGCTCCAGAGAAAAGACAACGAGCCCCATCAG 801

.||||| ||||||||||||||||||||||||||||||||||||||||

NbINO2 758 TATCATATTAATTTTTTCAGCTCCAGAGAAAAGACAACGAGCCCCATCAG 807

NbINO1 802 CTTATAACTGCTTTATCAAGTAATTATTAGATTGTTTATATGAGTTTGAG 851

|||||||||||||||||||||||||.|| |||||||.

NbINO2 808 CTTATAACTGCTTTATCAAGTAATTTTT--------------AGTTTGAA 843

NbINO1 852 AATTA--AGACTCATAATTAACATTAGTTTATAATACTTTACTAACATTA 899

.|||| ||||||||||||||||||||||||||.||||.|||||||||||

NbINO2 844 CATTAAGAGACTCATAATTAACATTAGTTTATAGTACTGTACTAACATTA 893

NbINO1 900 TTTATGTTTTACAGAGAAGAGATCAAGAGGCTAAAGACCATATACCCCAA 949

|||.||||||||||||||||||||||||||||||||||||||||||||||

NbINO2 894 TTTGTGTTTTACAGAGAAGAGATCAAGAGGCTAAAGACCATATACCCCAA 943

NbINO1 950 CATGACTCACAAGCAAGCTTTCAGTACGGCAGCAAAAAATGTGAGTTGAA 999

||||||||||||||||||.||||||||.|||||||||||||||||||.||

NbINO2 944 CATGACTCACAAGCAAGCCTTCAGTACCGCAGCAAAAAATGTGAGTTAAA 993

NbINO1 1000 TTTATT----CAAATGTTAATTATATGAACAAC-AAAACTTCTCTAAGCA 1044

|.|||| .|||||||.|| ||.||||||| |||||||.|||.||

NbINO2 994 TATATTTAAATAAATGTTTAT--TACGAACAACAAAAACTTTTCTTAG-- 1039

NbINO1 1045 AAATCTTATACGTATTCCAAAATTTGATTGGTTGTGTGTTCAGTGGGCCC 1094

.||||||||.|.|||||.|.|.||||||||||||

NbINO2 1040 ----------------TCAAAATTTTACTGGTTTTATATTCAGTGGGCCC 1073

NbINO1 1095 ACTTTCCACCAAGTCAGCATAGAGAAGATAGAGAAAGCTGTAGCCTGGGA 1144

||||||||||||||||.|||||||.|||||||||||||||||||||.|||

NbINO2 1074 ACTTTCCACCAAGTCAACATAGAGGAGATAGAGAAAGCTGTAGCCTAGGA 1123

NbINO1 1145 GACAGAAAGATGCCAAAG 1162

||||||||||||||||||

NbINO2 1124 GACAGAAAGATGCCAAAG 1141
