# Supplementary material for: Conservation of the role of INNER NO OUTER in development of unitegmic ovules of the Solanaceae despite a divergence in protein function
Source: BMC Plant Biol. 2016 Jun 27;16:143. doi: 10.1186/s12870-016-0835-z (PMC4924249; doi:10.1186/s12870-016-0835-z)
Supplement: Additional file 6: Table S2. — Results of VIGS experiments showing the number of plants and carpels analyzed. (DOCX 64 kb) [file 12870_2016_835_MOESM6_ESM.docx]

**Table S2**: Results of VIGS experiments showing the number of plants and carpels infiltrated and analyzed. Counts indicate number of aberrant ovules per carpel examined from experimental and control VIGS plants.

|  | **Plant #** | **# of aberrant ovules per carpel** |
| --- | --- | --- |
| *Experiment 1* | 1 | 8 (Fig. 3b), 3, 22, 16 |
|  | 2 | 8, 5, 7, 4 |
|  | 3 | Aberrant ovules observed, not counted. |
| *Experiment 2* | 1 | Aberrant ovules observed, not counted. |
|  | 2 | 16, 1, 2, 27 |
|  | 3 | 23, 20, 12 |
|  | 4 | 4, 6, 1 |
| *Experiment 3* | 1 | 3, 1, 2 |
|  | 2 | 1, 2, 2, 3 |
|  | 3 | 6, 6, 2, 2 |
|  | 4 | 15, 1, 4, 4, 3 |
|  |  |  |
| *Control 1* | 1 | 0, 0, 0, 0 |
|  | 2 | 0, 0 |
|  | 3 | 0 |
| *Control 2* | 1 | 0, 0 |
|  | 2 | 0, 0 |
| *Control 3* | 1 | 0, 0 |
|  | 2 | 0, 0, 0 |
|  | 3 | 0 |
